# Empirical Modeling of COVID-19 Evolution with High/Direct Impact on Public Health and Risk Assessment

**DOI:** 10.3390/ijerph19063707

**Published:** 2022-03-20

**Authors:** Noureddine Ouerfelli, Narcisa Vrinceanu, Diana Coman, Adriana Lavinia Cioca

**Affiliations:** 1 Institut Supérieur des Technologies Médicales de Tunis, Laboratoire de Biophysique et Technologies Médicales, Université de Tunis El Manar, Tunis 1006, Tunisia; nouerfelli@yahoo.fr; 2 Department of Industrial Machinery and Equipment, Lucian Blaga University of Sibiu, 550024 Sibiu, Romania; diana.coman@ulbsibiu.ro; 3 Independent Researcher, 557260 Sibiu, Romania; adriana.cioca@gmail.com

**Keywords:** COVID-19, empirical modeling, accelerated spread, delayed spread, mortality, Romania

## Abstract

This report develops a conceivable mathematical model for the transmission and spread of COVID-19 in Romania. Understanding the early spread dynamics of the infection and evaluating the effectiveness of control measures in the first wave of infection is crucial for assessing and evaluating the potential for sustained transmission occurring in the second wave. The main aim of the study was to emphasize the impact of control measures and the rate of case detection in slowing the spread of the disease. Non pharmaceutical control interventions include government actions, public reactions, and other measures. The methodology consists of an empirical model, taking into consideration the generic framework of the Stockholm Environment Institute (SEI) Epidemic–Macroeconomic Model, and incorporates the effect of interventions through a multivalued parameter, a stepwise constant varying during different phases of the interventions designed to capture their impact on the model. The model is mathematically consistent and presents various simulation results using best-estimated parameter values. The model can be easily updated later in response to real-world alterations, for example, the easing of restrictions. We hope that our simulation results may guide local authorities to make timely, correct decisions for public health and risk assessment.

## 1. Introduction

Coronavirus infections in humans were first identified in the 1960s [1,2,3,4,5]. The majority of coronavirus infections are respiratory, meaning they primarily affect the upper respiratory tract and lungs [5]. There are seven identified and reported coronaviruses, including 229E, NL63, OC43, HKU1, SARS-CoV, MERS-CoV, and SARS-CoV-2, that infect humans. Coronaviruses are zoonotic, meaning they primarily/basically infect animals and later gained the ability to infect humans [6,7]. Globally, the SARS-CoV pandemic came to the scene in 2002–2003 and led to 8000 infections in humans, with more than 770 deaths across 27 countries [8,9,10,11]. Accordingly, detection and controlling of such infection-based disease outbreaks have been a major concern in public health [7,12,13,14,15]. The novel 2019 severe acute respiratory syndrome coronavirus 2 (SARS-CoV-2) that is responsible for the ongoing COVID-19 pandemic has spread worldwide, causing a significant number of deaths. It is new and unlike the previous known virus-induced diseases. Data collected worldwide have indicated that older adults, particularly those with serious underlying health conditions, are at higher risk that younger persons for severe COVID-19-associated illness and death (CDC, 2020). As of 12 March 2020, COVID-19 had been confirmed in 125,048 people worldwide and carried a mortality rate of approximately 3.7% compared with a mortality rate of less than 1% for influenza. The development of a vaccine against SARS-CoV-2 is a cornerstone in the prevention of COVID-19 spread, but non pharmaceutical preventive measures are also vital. The non pharmaceutical preventive measures include frequent hand washing, use of face masks, cleaning and ventilation of indoors environments, social distancing, and successful mathematical models that could predict the speed of disease transmission and the efficacy of interventions [16,17]. There is a significant research effort, including mathematical modeling, to understand the characteristics and the epidemiological dynamics of the virus and its COVID-19 disease. Due to its novelty, the research is often likely to produce results only on specific aspects of the disease, providing only partial answers to research questions, or collect evidence for formulating a hypothesis yet to be tested. The continuously increasing number of COVID-19 cases necessitates sharing even such partial results as soon as they are available in order to facilitate the advancement of the research on this disease. While eventually a more comprehensive picture of both the virus and the disease will emerge, even incomplete but timely and scientifically based information will help authorities make sound decisions on the course of action during the epidemic [18,19,20]. Since SARS-CoV2 causes a contagious respiratory infection, the second wave started during the winter. Accordingly, a reliable predictive mathematical model is urgently needed to support the efforts in our battle against SARS-CoV2. Since the first reported case during the first wave, all countries were afraid and expecting the 2019 novel coronavirus (2019-nCoV) outbreak would lead to a large number of deaths. Every day estimates of the number of cases and deaths are provided by the authorities in all countries and distributed worldwide. From these data, the fragility of health systems in developing countries was uncovered. There are official data available for Romania to make empirical modeling of the pandemic transmission of COVID-19. The first reported case of COVID-19 in Romania was announced on 15 February 2020, and the first death was reported on 8 March 2020. At that time, the overall number of infected was estimated to be around 36.The number of cases was then increased exponentially to be more than 82,000 by the end of August 2020, with a similar increase in mortality to 3858 confirmed deaths during the same period. Undoubtedly, there are far more numbers of undetected cases due to the limited number of screening tests, public hesitancy to report, and, even worse, the denial attitudes of some people. Since the onset of COVID-19 spread in Romania, the government has allocated isolation hospitals across the country and gradually placed a series of measures to prevent the disease from attaining widespread community transmission in attempts to flatten the rate of spread. Romania decided to lockdown the schools and universities on 10 March 2020 in response to the number of cases exceeding 100. A curfew was imposed, during which all public and private transportation was suspended. Air flights, social activities, worship meetings, and sports events were also suspended in the same period [7,21]. These activities were resumed partially towards the end of June 2020, with a continued gradual decline in the restrictions. There were efforts to fund many scientific projects aiming to improve the preventive measures, diagnostic procedures, and development of treatment for COVID-19. The ministry of health published and updated the Romanian protocol for the management of COVID-19 cases. After a short lag period, the outbreak started growing again. Some specialists expected a second wave of disease transmission that would pose a socioeconomic crisis and a huge burden on the public health of millions of people globally. From the transmission curve of the virus, the three phases of the COVID-19 pandemic in Romania can be distinguished. First, the latent growth phase, where the number of daily reported cases is almost constant from day to day, lasted from 15 February to 28 February (Table S1). The second phase of the pandemic corresponds to the accelerated growth phase and started on the 29 of February. The third phase, beginning on 19 June, corresponds to the delayed growth phase, where a time-dependent, exponentially decreasing transmission rate occurs due to major public interventions and social distancing measures [22,23,24,25,26,27,28,29,30,31,32,33,34,35,36,37,38,39,40,41,42,43,44,45,46,47,48,49,50]. Mathematical models have been proven to play a significant role in the study of infectious diseases [23]. They can provide deeper insight into the dynamics of the diseases’ spread and also suggest effective control strategies to help local public health authorities in the process of control and decision-making/risk management to protect populations and to end the crisis [24,25,34,35,36,37,38,39,40,41,42,43,44,45,46,47,48,49,50]. In the present work, as a semi-empirical study, the pandemic spread behavior will be modeled in simplified exponential form over time with three adjustable parameters in order to offer an accurate prediction and estimation, as well as to contribute to the improvement and the advancement of certain theories by proposing a particular solution to their systems by using theoretical equations. In other words, our study is focused on a conceivable mathematical model of official data concerning the impacts of COVID-19 on the quality of public health and the interrelationship between environmental health and quality of life. Our simulation results may guide local authorities to make timely, correct decisions for public health and risk assessment.

## 2. Reported Cases and Deaths Data

### 2.1. Data Scope

**Figure 1:**
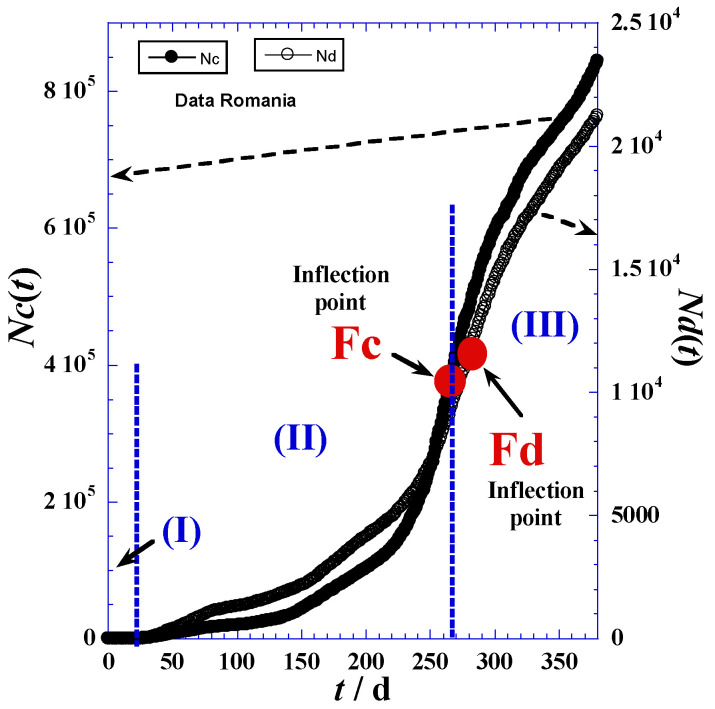
Total reported cases and deaths for the first 440 days of the pandemic in Romania.

The investigation data of reported cases and deaths attributed to COVID-19 in Romania are collected for a period of about twenty-one months (from 25 February 2020 to 11 December 2021) from three principal electronic resources. The first is the COVID-19 dashboard of the Johns Hopkins University [26], the second are the COVID-19 world meters [27], and the third is GitHub [28]. Data are given in Table 1 in the Supplementary Materials section and are depicted in Figure 1. In view of the adequate empirical expressions, we propose to divide the time range into three domains according to the different trends of curvatures shownin Figure 1. Table 1 indicates the three main phases: (I) latent phase, (II) accelerated phase, and (III) delayed phase.

Baldwin, 2020 [29], has considered that the two phases (0) and (I) in Table 1 constitute the pre-pandemic intervals and can be assigned as the two stages of Investigation and Recognition, while phases (II) and (III) constitute the pandemic intervals and can be assigned as the four stages of Initiation, Acceleration, Deceleration, and Preparation.

### 2.2. Delimitation of Phases’ Domains

The delineation of the three domains mainly depends on the accuracy in determination of the inflection point coordinates indicated in Figure 1 and Table 1, such as Fc(*t*_*c*1_,*N*_*c*1_) and Fd(*t*_*d*1_,*N*_*d*1_) for the reported daily cases and daily deaths, respectively. We note that the initial times (*t*_*c*0_) and (*t*_*d*0_) of phase (I) correspond to the day before the first non-null appearance of a new case (Table 2 and Table S1). On the other hand, the final times of the latent phase (I), which are the initial times (*t*_*c*1_) and (*t*_*d*1_) of the accelerated phase (II), are obtained by optimization techniques using least-square methods and nonlinear regression of the proposed model for the accelerated phase (II) presented in Section 3. However, the times (*t*_*c*1_) and (*t*_*d*1_) of the inflection points (Fc) and (Fd) in Figure 1 can be determined by two techniques—the derivation method and tangent method—which will be detailed below.

#### Derivation Method

The ideal and precise technique consists in modeling by smoothing some small portions of the curve with similar curvatures using simple nonlinear regression, such as a low-degree polynomial, and then making a derivative of each part (Equation (1)) with the precaution of ensuring continuity and derivability for each boundary.

Then, the derivative function reaches its maximum exactly at the inflection times (*t*_*c*1_) and (*t*_*d*1_).

(1) $$n_{i}\left(t\right)=\frac{dN_{i}\left(t\right)}{dt}$$

However, due to the irregularities of curvature, we can encounter some difficulties in the partial modeling. In this situation, we can use the relative variation for a very small interval of time (Equation (2)) or simplify the daily reported case given from the provided data.

(2) $$n_{i}\left(t\right)=\frac{\Delta N_{i}\left(t\right)}{\Delta t}$$

**Figure 2:**
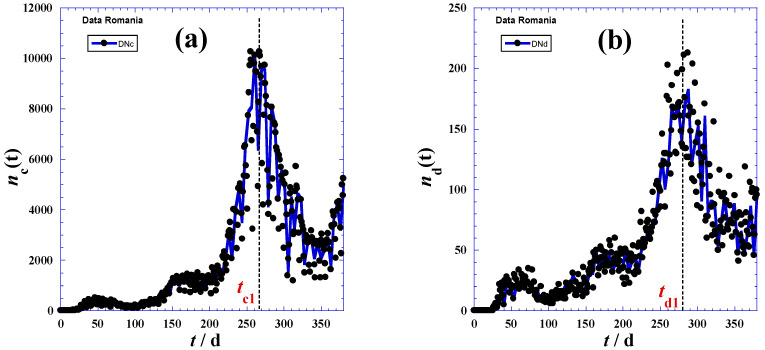
New daily reported cases (**a**) and new daily reported deaths (**b**) for the first 380 days of the pandemic in Romania.

In practice, we can also approximately determine the inflection times (*t*_*c*1_) and (*t*_*d*1_) when the daily reported cases reach the maximum of the smoothed peak (Figure 2). Indeed, the inflection points (Fc) and (Fd) occur at the daily highest cases *t*_*c*1_ and the daily highest deaths *t*_*d*1_. Consequently, corresponding coordinates: *N*_*c*1_ = *N*_*c*_(*t* = *t*_*c*1_) and *N*_*d*1_ = *N*_*d*_(*t* = *t*_*d*1_), respectively, are available (Table 1).

## 3. Accelerated Phase Modeling

Once the two inflection times (*t*_*c*1_) and (*t*_*d*1_), as the second boundary of the accelerated phase (II) are determined, the first limit of times (*t_c_*) and (*t*_*d*_) can be adequately recognized only by nonlinear regression by optimizing the standard deviation (*σ*) and the relative error (*E_rel_*) between the experimental values (Table S1) and the values estimated by the proposed model.

However, due to the pseudo-Gaussian shape of the derivative function d*N*(*t*)/d(*t*) plotted in Figure 2, the following expressions with three independent adjustable parameters for the reported cumulative cases *N_c_(t)* and the recorded cumulative deaths *N_d_(t)* in the accelerated phase (II) were suggested:

(3) $$N_{c}\left(t\right)=N_{c0}\left(e^{\frac{\left(t-t_{c}\right)}{\tau_{c}}}-1\right)+\delta N_{c}$$

(4) $$N_{d}\left(t\right)=N_{d0}\left(e^{\frac{\left(t-t_{d}\right)}{\tau_{d}}}-1\right)+\delta N_{d}$$

where increments of (*δN_c_*) and (*δN_d_*) are reliant on parameters and can be adjusted to the values of the reported cases *N_c_*(*t*_*c*_) and the deaths *N_d_*(*t*_*d*_) at the start of the accelerated phase (II), with optimization being the main preferred mean irrespective of the existence of a slight difference from the experimental values shown in Table S1.

(5) $$\delta N_{c}\;\approx \;N_{c}\left(t_{c}\right)$$

and

(6) $$\delta N_{d}\;\approx \;N_{d}\left(t_{d}\right)$$

The optimal values reported for the adjustable parameters for the accelerated phase (II) cases and deaths as determined by nonlinear regressions for Equations (3) and (4) are identifiable from Table 2. All terminology has been defined above and applied to Equations (1)–(8).

We note that (*A_c_*_0_) and (*A_d_*_0_) denote case activity and death activity, respectively, expressed as follows:

(7) $$A_{c0}=\frac{N_{c0}}{\tau_{c}}$$

and

(8) $$A_{d0}=\frac{N_{d0}}{\tau_{d}}$$

**Figure 3:**
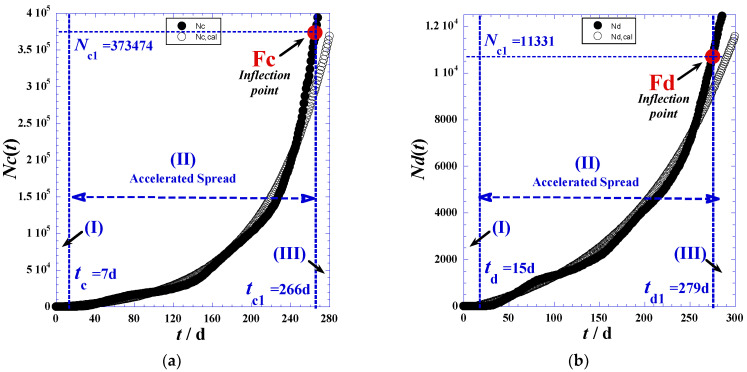
(**a**) The total reported cases *N_c_*(*t*) for the first 200 days of the pandemic in Romania, the accelerated phase (II) using Equation (3). (**b**) The total reported deaths *N_d_*(*t*) for the first 200 days of the pandemic in Romania, the accelerated phase (II) using Equation (4).

Figure 3 shows an excellent agreement between the experimental values and the estimated ones in the accelerated phase (II), while the discrepancy observed in the delayed phase (III) prompts us to slightly modify the model of Equation (3) to predict this slower phase.

Nevertheless, there are studies which stated that “the spread of COVID-19 is not going to follow an exponential curve and serious errors will follow if analysts believe it would [30]. The number of new cases rises rapidly, peaks, and then declines. It’s called the epidemiological curve. It’s not a theory or hypothesis”. This statement is real/true if the phenomenon is described with a simple exponential form. There are several causes that interfere together which give such complicated propagation, which necessitates expression by an exponential function whose argument is a non-simple function of time. Given that what we are proposing is empirical, we have suggested the simplest possible form (Equation (3)). In fact, Figure S4 justifies our choice, where the linearity of the logarithm can be seen in a wide range of time in the accelerated phase (II), which is the subject of the proposed model.

Furthermore, the peak height (Figure 2a, Equation (9)) defined as the maximum of the derivative function of *N_c_*(*t*_*c*1_) occurring at the highest day (*t*_*c*1_) and an inflection point (Fc) for the *N*_*c*_(*t*)-curve (Figure 1)—indicates that containment efforts are ineffective and interventions are minimal [30], and the curve takes on different shapes depending on the virus’s infection rate and the health system’s capacity [31]. 

The maximum derivative function of *N_c_*(*t*_c1_) which occurs at the highest day (*t*_c1_) coupled with inflection point (Fc) for the *N_c_*(*t*)-curve (Figure 1) causes the peak height (Figure 2a, Equation (9)), being an indication of very weak containment policies and negligible interventions [32,33]. The curve assumes diverse shapes, depending on the infection rate of the virus and the health system capacity [34].

(9) $$n_{c,max}\left(t=t_{c1}\right)=A_{c0}e^{\frac{\left(t_{c1}-t_{c}\right)}{\tau_{c}}}$$

## 4. Correlation between Reported Cases and Deaths

Considering the present work as an empirical investigation, this section will only introduce empirical comparisons in order to help theoreticians to invest in more details in their investigations of the applicable theoretical parameters.

As a first examination from Table 2, we can write the following inequalities:

(10) $$\{\begin{array}{c} t_{d}>t_{c}\\ N_{d0}<N_{c0}\\ \tau_{d}<\tau_{c} \end{array}$$

This must be considered as mathematically necessary criteria and fundamental limitations in Romanian optimization issues. We can also add the following derived parameters necessary for subsequent discussions and interpretations:

(11) $$\{\begin{array}{c} \Delta t=t_{d}-t_{dc}\\ \Delta N=N_{c0}-N_{d0}\\ \Delta \tau =\tau_{d}-\tau_{c} \end{array}$$

*A_d_*_0_ < *A_c_*_0_ (12)

**Figure 4:**
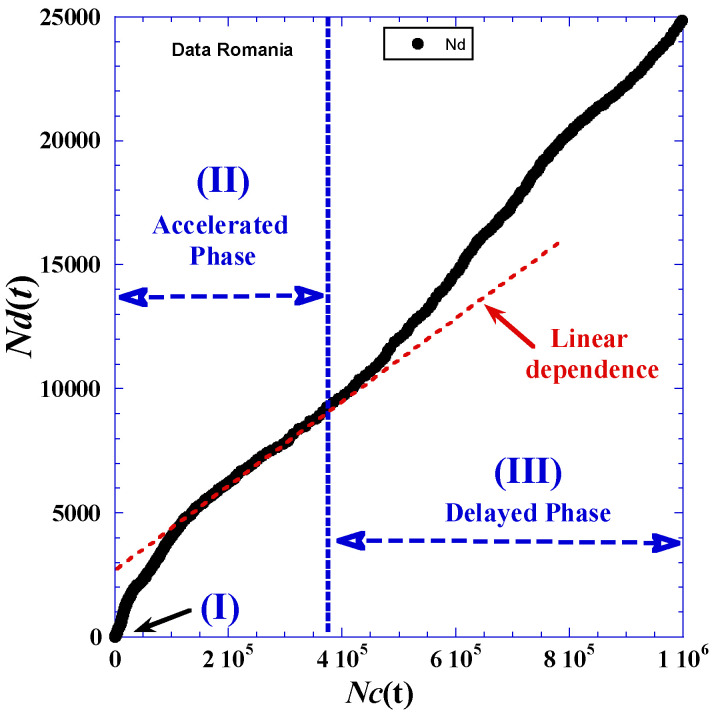
Cumulative deaths *N_d_*(*t*) versus the total reported cases *Nc*(*t*) for the first 350 days of the pandemic in Romania.

One of the ways to see the mutual correlation between the reported cumulative cases *N_c_*(*t*) and the cumulative deaths *N_d_*(*t*) is to eliminate the time-variable and plot *N_d_*(*t*) as a function of *N_c_*(*t*), like in Figure 4. We observe an interesting linear dependence in a domain stretched between the two accelerated (II) and delayed (III) phases. After that, the positive deviation to the linearity (with high slope value) indicates that each reported case’s phase always precedes in time the similar phase related to death cases.

Another manner of comparison consists of introducing the mortality rate *T*(*t*), expressed as follows:

(13) $$T\left(t\right)=\frac{N_{d}\left(t\right)}{N_{c}\left(t\right)}$$

**Figure 5:**
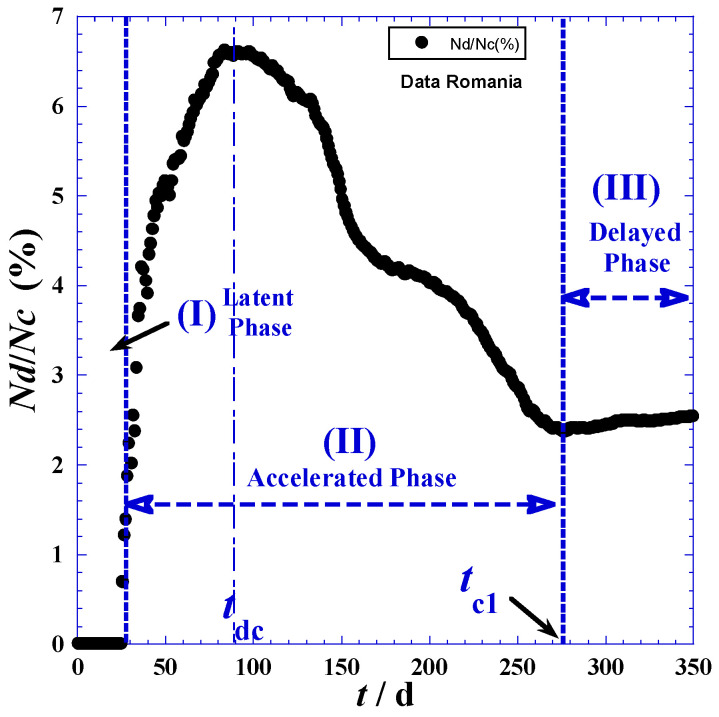
Mortality rate *T*(*t*) as a function of time for the first 350 days of the pandemic in Romania.

The plot of the mortality rate *T*(*t*) in percent is shown in Figure 5. Distinct behaviors of the three spread phases are observed. The maximum occurring in the accelerated phase at (*t = t_dc_*) of about 64 days is due mathematically to the sign conflict between the two logarithms *lnN_d_(t*) and *lnN_c_(t)*, which is clearly revealed in Figure S5. There is a benefit from this feature by following this variation over time from the beginning of the spread, and, when the mortality rate *T*(*t*) reaches the maximum, we can predict that the pandemic is preparing to move from the accelerated phase (I) to the delayed phase (II) (assuming there are no great changes in precautionary measures and the peoples’ behavior towards the pandemic.)

## 5. Prediction of Delayed Phase for Symmetric Behavior

To predict the delayed phase (III) from only the accelerated phase (II) data, we must consider as a first approximation that the kinetic progress of the COVID-19 pandemic is the same before and after the highest day (*t* = *t*_*c*1_). This symmetric behavior occurs when there are no changes in the pandemic environments, such as precautionary measures and peoples’ behavior toward the COVID-19 pandemic, etc. On the other hand, the symmetric behavior is translated mathematically by the fact that the inflection point (Fc) will be a center of symmetry of the curve in Figure 1.

Herein, there is a trial of predicting the delayed phase by transforming the proposed model by two assumptions. Subsequently, by following the boundary conditions, continuity and the derivability at the inflection point F_c_(*t* = *t*_*c*1_), the equation predicting the delayed phase (III) is expressed is as follows:

(14) $$N_{c}\left(t\right)=N_{c1}\left(2-e^{\frac{-\left(t-t_{c1}\right)}{\tau^{\prime }{}_{c}}}\right)$$

with

(15) $$\tau^{\prime }{}_{c}=\frac{\tau_{c}N_{c1}}{N_{c0}}e^{\frac{-\left(t_{c1}-t_{c}\right)}{\tau_{c}}}$$

**Figure 6:**
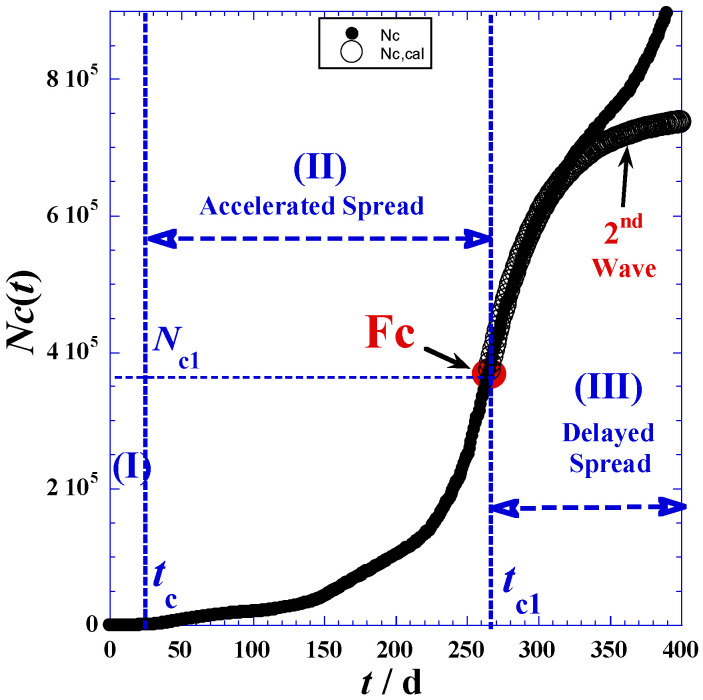
Total reported cases *Nc*(*t*) for the first 400 days for delayed phase (III) in symmetric behavior using Equation (14) and *τ_c_*’ = 46 days.

In this case, *τ*′_*c*_ = 37 days. The previous values are close to the (*τ*_*c*_) given in Table 2 for the accelerated phase (II). Therefore, we can conclude that in a reliable approximation, we can simplify the problem and put the value of (*τ*_*c*_) in Equation (14) in place of (*τ*′_*c*_) without any net imprecision (Figure 6). The discrepancy between experimental values and estimated ones within 320 days is due to the fact that the process of spread is not symmetric.

## 6. Prediction of Delayed Phase for Asymmetric Behavior

Generally, and for real situations, we cannot observe the symmetric behavior previously mentioned due to the instantaneous change of peoples’ behaviors and authorities’ decisions. So, respecting the continuity and the derivability on the highest day (*t* = *t*_*c*1_) occurring at the inflection point Fc (*t* = *t*_*c*1_), the equation predicting the delayed phase (III) becomes expressed as follows:

(16) $$N_{c}\left(t\right)=N_{c1}+\frac{\tau^{\prime }{}_{c}N_{c0}}{\tau_{c}}\left(1-e^{\frac{-\left(t-t_{c1}\right)}{\tau^{\prime }{}_{c}}}\right)e^{\frac{\left(t_{c1}-t_{c}\right)}{\tau_{c}}}$$

Here, only one adjustable parameter (*τ*′_*c*_) is needed to be estimated using optimizations techniques. The downside of this situation is that we cannot apply any nonlinear regression if we do not have enough data points after the highest day (*t* = *t*_*c*1_). However, a successful prediction should also be in agreement with the limiting value (*Nc*_∞_) of the reported cumulative case at the end of the COVID-19 pandemic (Equation (17)).

(17) $$N_{c\infty }=N_{c1}+\frac{\tau^{\prime }{}_{c}N_{c0}}{\tau_{c}}e^{\frac{\left(t_{c1}-t_{c}\right)}{\tau_{c}}}$$

**Figure 7:**
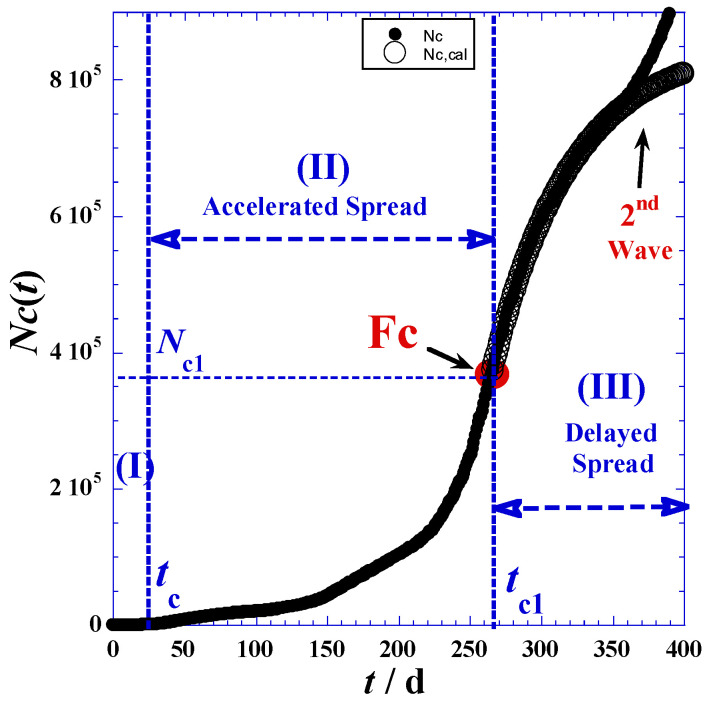
Total reported cases *Nc*(*t*) of the first 400 days for delayed phase (III) in asymmetric behavior using Equation (16) with *τ_c_*′ = 56 days and *N_c∞_* = 753,500.

Figure 7 shows net improvement relative to the symmetric prediction using in Equation (17) an optimal value (*τ*′_*c*_ = 56 days) determined by the least-square method of nonlinear regression.

Starting at 400 days, we can notice the emergence of a new wave with a larger amplitude than the previous one.

## 7. Results and Discussion

In the case of an epidemic, prediction at an initial stage plays a pivotal role to control the epidemic. Government agencies and public health organizations can prepare accordingly as per the prediction. Indeed, mathematical models play a key role in generating quantitative information in the field of epidemiology by providing important guidelines for outbreak management for decision makers. To improve the forecast accuracy and investigate the spread of SARS-CoV-2 during the second wave, we established an empirical mathematical model in order to numerically estimate the spread of SARS-CoV-2 in Romania. This study is trying to do what the American president, Theodore Roosevelt, said: “The more you know about the past, the better prepared you are for the future”. This model is linking the Romanian governmental efforts to limit COVID-19 spread in Romania and to reduce mortality in infected cases with the real-life situation of the first wave. Estimating and predicting the number of people affected by COVID-19 is crucial in deciding which public health policies to follow. After the appearance of the first case of COVID-19 infection in Romania on 15 February, there were no public restrictions to prevent the disease spread. The Romanian government depended mainly on spreading awareness about the importance of wearing masks, hand hygiene, and social distancing. This action was quite enough until the end of February 2020, the end of the latent phase.

This analysis declares that Romania responded adequately to the beginning of the accelerated phase. After about ten days after the onset of the accelerated phase, Romania started gradually increasing public lockdown procedures. The accelerated phase continued for about 110 days despite the governmental actions, which may reflect the lack of public commitment to the imposed restrictions. The higher mortality rates observed in Romania cannot be attributed to a defect in the Romanian health system, as there was no curative treatment for SARS-CoV-2 up until now. These higher mortality rates may be explained by the delay in seeking medical aid due to the fear of stigma. This analytic model can lead to better management for the expected second wave of COVID-19 infection in Romania. To shorten the duration of the accelerated phase in the second wave, we suggest, based on this model, to hasten the lockdown procedure in response to any increase in the daily number of cases. Emphasis on implementing the established restrictions and on imposing sanctions on nonconformists may also abbreviate the accelerated phase.

The Romanian authorities should encourage people to seek medical advice as early as they feel any symptom of COVID-19 infection. This may decrease the mortality rates all over the world, as earlier medical intervention was shown to abate the mortality rates in SARS-CoV-2 infection cases [36,37,38,39,40,41,42,43]. The authorities in different countries carry out mortality counts. The mortality reported in each country can be used to create an index of the number of actual cases at a given time. The specificity of whether or not COVID-19 causes mortality is rapid, affecting the number of actual cases. The number of days between the declaration of illness and death varies between 12 and 18 days. The mortality rate up to10 October reached 5.8%, which is one of the highest reported death values worldwide. The pessimistic scenario predicted 22,320 infected people, and the most optimistic predicted 744 (which is the number of reported cases on 12 April 2020). Modeling the occurrence of COVID-19 cases is critical to assess the impact of policies to prevent the spread of the virus. The main objective of this study is the estimation of the average number of infections one case can generate throughout the infectious period. It is the basic reproduction number of an infectious agent.

## 8. Conclusions

The current mathematical effort uses the dataset to produce an estimate of the true number of infection cases in Romania, based on the 23-day effect from infection to death. The data includes the cumulative number of reported cases. Two deterministic compartmental models based on the clinical progression of the disease and the epidemiological status of the individuals have been proposed. The SEIR model of dengue fever studies the disease transmission based on the Susceptible–Exposed–Infectious–Removed (SEIR) model, including an asymptomatic transmission rate. Four categories are present: susceptible, exposed, infectious, and recovered. The cumulative number of reported symptomatic infectious cases at time *t*, denoted by *N_c_(t*), is computed. Afterward, development and comparison of numerical simulations with data were performed. To give physical meaning to the three parameters in our suggested model for future work, probable causal correlation with factors such as infected, recovered, hospitalized, serious cases, etc. will be investigated.

Future modifications can be easily accommodated by updating the model (for example, the easing of some restrictions.)

## Figures and Tables

**Table 1:** Different spread phases and identification of the accelerated phase for the reported cases and deaths.

Phase 0		Phase I		Phase II		Phase III
Reported cases						
Absence	*t* = *t*_*c*0_	Latent	*t* = *t*_*c*_	Accelerated	*t* = *t*_*c*1_	Delayed
Deaths						
Absence	*t* = *t*_*d*0_	Latent	*t* = *t*_*d*_	Accelerated	*t* = *t*_*d*1_	Delayed

**Table 2:** Different spread phases and identification of the accelerated phases.

*t*_*c*0_	*t_c_*	*t_c_*_1_	$\tau_{c}$	$N_{c0}$	*Erel*	*σ*	*N_c_*_1_	*A_c_*_0_
0	7	266	67.92	6745	7.85%	13,693	373,474	99.31
*t*_*d*0_	*t_d_*	*t_d_*_1_	$\tau_{d}$	$N_{d0}$	*Erel*	*σ*	*N_d_*_1_	*A_d_*_0_
25	15	279	121.88	1135	9.78%	404.3	11,331	9.312

## Data Availability

All data supporting reported results can be found in the References part.

## Supplementary Materials

The following supporting information can be downloaded at: https://www.mdpi.com/article/10.3390/ijerph19063707/s1, Table S1. Data of Reported cases and deaths of Covid-19 for Romania for about twenty-one months (from 25 February 2020 to 11 December 2021). Figure S1. Total Reported Cases for the First 440 days of the Pandemic in Romania. Figure S2. New daily reported cases (a) and new daily deaths (b) for the eight months of the pandemic in Romania (Reported from original data without smoothing of duplicated values). Scheme S1. Flowchart for Adjustment the Duplicate Data in Romania Excel Sheet. Figure S3. (a) Graphical determination of the inflection point Fc(*t*_*c*1_,*N_c_*_1_) related the total reported cases *N_c_*(*t*) for the first 350 days in Romania. (b) Graphical determination of the inflection point Fd(*t**_d_*_1_,*N_d_*_1_) related the total death cases *N_d_*(*t*) for the first 350 days in Romania. Figure S4. Second derivative function (d2Nc.dt2) related the total death cases *N_c_*(*t*) for the first 400 days in Romania. Figure S5. Natural logarithm of total reported cases ln*N_c_*(*t*) with time in the accelerated phase (II). Figure S6. Difference between logarithms of the derivative functions of the total reported cases and deaths; to confirm the global maximum occurring InNc.Nd-Curve (Figure 5).
