# Supplementary material for: Empirical Modeling of COVID-19 Evolution with High/Direct Impact on Public Health and Risk Assessment
Source: Int J Environ Res Public Health. 2022 Mar 20;19(6):3707. doi: 10.3390/ijerph19063707 (PMC8948883; doi:10.3390/ijerph19063707)
Supplement: Supplementary file 1 [file ijerph-19-03707-s001.zip › ijerph-1545203-supplementary.pdf]

Supplementary Materials

Empirical Modeling of COVID-19 Evolution with High/Direct Impact on Public Health and Risk Assessment

Noureddine Ouerfelli[1], Narcisa Vrinceanu[2]*, Diana Coman[2] and Adriana Lavinia Cioca[3]

1 Institut Supérieur des Technologies Médicales de Tunis, Laboratoire de Biophysique et Technologies Médicales, Université de Tunis El Manar, Tunis , 1006, Tunisia; nouerfelli@yahoo.fr
2 Department of IndustrialMachinery and Equipment, Lucian Blaga University of Sibiu, 10 VictorieiBoulevard , 550024 Sibiu, Romania; diana.coman@ulbsibiu.ro
3 Independent researcher, CMI Dr. Cioca Adriana-Lavinia , 22 Dorobantilor Street , Sibiu, 557260 Sibiu, Romania; adriana.cioca@gmail.com
* Correspondence: vrinceanu.narcisai@ulbsibiu.ro; Tel.: +40-721-428-641

1S. Data Collection

We have collected data from electronic sources [A,B,C], for about 440 days for Romania (from February 25, 2020 to May 09, 2021).

[A] COVID-19 dashboard, Center for Systems Science and Engineering (CSSE), Johns Hopkins University (JHU) 2020. https://systems.jhu.edu/
[B] COVID-19 Coronavirus Pandemic, https://www.worldometers.info/coronavirus/
[C] Github, Our World in Data, https://github.com/owid/covid-19-data/tree/master/public/data
https://gisanddata.maps.arcgis.com/apps/opsdashboard/index.html#/bda7594740fd40299423467b48e9ecf6
https://www.statista.com/statistics/1102614/romania.coronavirus.cases/

Table S1.Data of Reported cases and deaths of Covid-19 for Romania for about twenty-one months (from February 25, 2020 to Dec.11, 2021).

Date	Time (t).d	Total cases	New cases	Total deaths	New deaths	Date	Time (t).d	Total cases	New cases	Total deaths	New deaths
24.02.2020	0	0	0	0	0	2021.01.18	328	695153	1509	17271	50
25.02.2020	0	0	0	0	0	2021.01.19	329	697898	2745	17369	98
26.02.2020	1	1	1	0	0	2021.01.20	330	700898	3000	17485	116
27.02.2020	2	1	0	0	0	2021.01.21	331	703776	2878	17554	69
28.02.2020	3	3	2	0	0	2021.01.22	332	706475	2699	17628	74
29.02.2020	4	3	0	0	0	2021.01.23	333	709194	2719	17722	94
01.03.2020	5	3	0	0	0	2021.01.24	334	711010	1816	17776	54
02.03.2020	6	3	0	0	0	2021.01.25	335	712561	1551	17841	65
03.03.2020	7	3	0	0	0	2021.01.26	336	715438	2877	17938	97
04.03.2020	8	4	1	0	0	2021.01.27	337	718612	3174	18015	77
05.03.2020	9	6	2	0	0	2021.01.28	338	721513	2901	18105	90
06.03.2020	10	9	3	0	0	2021.01.29	339	724250	2737	18196	91
07.03.2020	11	9	0	0	0	2021.01.30	340	726918	2668	18264	68
08.03.2020	12	15	6	0	0	2021.01.31	341	728743	1825	18335	71
09.03.2020	13	15	0	0	0	2021.02.01	342	730056	1313	18402	67
10.03.2020	14	25	10	0	0	2021.02.02	343	732732	2676	18513	111
11.03.2020	15	45	20	0	0	2021.02.03	344	735484	2752	18600	87
12.03.2020	16	49	4	0	0	2021.02.04	345	738152	2668	18675	75
13.03.2020	17	89	40	0	0	2021.02.05	346	740732	2580	18748	73
14.03.2020	18	123	34	0	0	2021.02.06	347	743343	2611	18809	61
15.03.2020	19	131	8	0	0	2021.02.07	348	745318	1975	18881	72

16.03.2020	20	158	27	0	0	2021.02.08	349	746637	1319	18961	80
17.03.2020	21	184	26	0	0	2021.02.09	350	749434	2797	19056	95
18.03.2020	22	260	76	0	0	2021.02.10	351	752482	3048	19135	79
19.03.2020	23	277	17	0	0	2021.02.11	352	755126	2644	19200	65
20.03.2020	24	308	31	0	0	2021.02.12	353	757676	2550	19277	77
21.03.2020	25	367	59	0	0	2021.02.13	354	760091	2415	19325	48
22.03.2020	26	433	66	3	3	2021.02.14	355	761963	1872	19366	41
23.03.2020	27	576	143	7	4	2021.02.15	356	763294	1331	19445	79
24.03.2020	28	794	218	11	4	2021.02.16	357	765970	2676	19526	81
25.03.2020	29	906	112	17	6	2021.02.17	358	768785	2815	19588	62
26.03.2020	30	1029	123	23	6	2021.02.18	359	771843	3058	19659	71
27.03.2020	31	1292	263	26	3	2021.02.19	360	774555	2712	19738	79
28.03.2020	32	1452	160	37	11	2021.02.20	361	777276	2721	19795	57
29.03.2020	33	1815	363	43	6	2021.02.21	362	779695	2419	19847	52
30.03.2020	34	2109	294	65	22	2021.02.22	363	781329	1634	19894	47
31.03.2020	35	2245	136	82	17	2021.02.23	364	784711	3382	20013	119
01.04.2020	36	2460	215	92	10	2021.02.24	365	788048	3337	20086	73
02.04.2020	37	2738	278	115	23	2021.02.25	366	791971	3923	20167	81
03.04.2020	38	3183	445	133	18	2021.02.26	367*	795732	3761	20233	66
04.04.2020	39	3613	430	146	13	2021.02.27	368	799164	3432	20287	54
05.04.2020	40	3864	251	151	5	2021.02.28	369	801994	2830	20350	63
06.04.2020	41	4057	193	176	25	2021.03.01	370	804090	2096	20403	53
07.04.2020	42	4417	360	197	21	2021.03.02	371	808040	3950	20509	106
08.04.2020	43	4761	344	220	23	2021.03.03	372	812318	4278	20586	77
09.04.2020	44	5202	441	248	28	2021.03.04	373	816589	4271	20684	98
10.04.2020	45	5467	265	270	22	2021.03.05	374	820931	4342	20785	101
11.04.2020	46	5990	523	291	21	2021.03.06	375	824995	4064	20854	69
12.04.2020	47	6300	310	316	25	2021.03.07	376	828283	3288	20900	46
13.04.2020	48	6633	333	331	15	2021.03.08	377	830563	2280	20963	63
14.04.2020	49	6879	246	351	20	2021.03.09	378	835552	4989	21056	93
15.04.2020	50	7216	337	372	21	2021.03.10	379	840116	4564	21156	100
16.04.2020	51	7707	491	392	20	2021.03.11	380	845352	5236	21252	96
17.04.2020	52	8067	360	411	19	2021.03.12	381	850362	5010	21360	108
18.04.2020	53	8418	351	421	10	2021.03.13	382	855326	4964	21439	79
19.04.2020	54	8746	328	451	30	2021.03.14	383	859709	4383	21483	44
20.04.2020	55	8936	190	478	27	2021.03.15	384	862681	2972	21565	82
21.04.2020	56	9242	306	498	20	2021.03.16	385	868799	6118	21698	133
22.04.2020	57	9710	468	524	26	2021.03.17	386	874985	6186	21787	89
23.04.2020	58	10096	386	545	21	2021.03.18	387	881159	6174	21877	90
24.04.2020	59	10417	321	567	22	2021.03.19	388	886752	5593	22020	143
25.04.2020	60	10635	218	601	34	2021.03.20	389	892848	6096	22132	112
26.04.2020	61	11036	401	619	18	2021.03.21	390	897115	4267	22208	76
27.04.2020	62	11339	303	641	22	2021.03.22	391	900858	3743	22268	60
28.04.2020	63	11616	277	663	22	2021.03.23	392	907007	6149	22442	174
29.04.2020	64	11978	362	693	30	2021.03.24	393	913143	6136	22579	137
30.04.2020	65	12240	262	717	24	2021.03.25	394	919794	6651	22719	140
01.05.2020	66	12567	327	744	27	2021.03.26	395	926310	6516	22835	116
02.05.2020	67	12732	165	771	27	2021.03.27	396	932179	5869	22997	162
03.05.2020	68	13163	431	790	19	2021.03.28	397	936618	4439	23114	117
04.05.2020	69	13512	349	818	28	2021.03.29	398	940443	3825	23234	120
05.05.2020	70	13837	325	841	23	2021.03.30	399	946647	6204	23409	175
06.05.2020	71	14107	270	864	23	2021.03.31	400	952803	6156	23538	129
07.05.2020	72	14499	392	888	24	2021.04.01	401	958918	6115	23674	136
08.05.2020	73	14811	312	923	35	2021.04.02	402	964726	5808	23819	145
09.05.2020	74	15131	320	939	16	2021.04.03	403	970224	5498	23973	154
10.05.2020	75	15362	231	961	22	2021.04.04	404	974375	4151	24070	97
11.05.2020	76	15588	226	982	21	2021.04.05	405	977986	3611	24190	120

12.05.2020	77	15778	190	1002	20	2021.04.06	406	983217	5231	24386	196
13.05.2020	78	16002	224	1036	34	2021.04.07	407	988624	5407	24561	175
14.05.2020	79	16247	245	1053	17	2021.04.08	408	993613	4989	24733	172
15.05.2020	80	16437	190	1070	17	2021.04.09	409	998555	4942	24867	134
16.05.2020	81	16704	267	1094	24	2021.04.10	410	1002865	4310	25006	139
17.05.2020	82	16871	167	1107	13	2021.04.11	411	1006167	3302	25135	129
18.05.2020	83	17036	165	1120	13	2021.04.12	412	1008490	2323	25248	113
19.05.2020	84	17191	155	1137	17	2021.04.13	413	1012373	3883	25441	193
20.05.2020	85	17387	196	1147	10	2021.04.14	414	1016449	4076	25605	164
21.05.2020	86	17585	198	1156	9	2021.04.15	415	1020301	3852	25800	195
22.05.2020	87	17712	127	1166	10	2021.04.16	416	1023565	3264	25937	137
23.05.2020	88	17857	145	1176	10	2021.04.17	417	1027039	3474	26072	135
24.05.2020	89	18070	213	1185	9	2021.04.18	418	1029304	2265	26232	160
25.05.2020	90	18283	213	1205	20	2021.04.19	419	1031072	1768	26381	149
26.05.2020	91	18429	146	1216	11	2021.04.20	420	1034003	2931	26618	237
27.05.2020	92	18594	165	1227	11	2021.04.21	421	1037009	3006	26793	175
28.05.2020	93	18791	197	1235	8	2021.04.22	422	1039998	2989	26943	150
29.05.2020	94	18982	191	1248	13	2021.04.23	423	1042521	2523	27113	170
30.05.2020	95	19133	151	1259	11	2021.04.24	424	1044722	2201	27267	154
31.05.2020	96	19257	124	1266	7	2021.04.25	425	1046264	1542	27394	127
01.06.2020	97	19398	141	1276	10	2021.04.26	426	1047520	1256	27511	117
02.06.2020	98	19517	119	1288	12	2021.04.27	427	1049539	2019	27683	172
03.06.2020	99	19669	152	1296	8	2021.04.28	428	1051779	2240	27833	150
04.06.2020	100	19907	238	1305	9	2021.04.29	429	1053629	1850	27971	138
05.06.2020	101	20103	196	1316	11	2021.04.30	430	1055265	1636	28109	138
06.06.2020	102	20290	187	1322	6	2021.05.01	431	1056572	1307	28194	85
07.06.2020	103	20479	189	1333	11	2021.05.02	432	1057655	1083	28282	88
08.06.2020	104	20604	125	1339	6	2021.05.03	433	1058337	682	28380	98
09.06.2020	105	20749	145	1354	15	2021.05.04	434	1059331	994	28474	94
10.06.2020	106	20945	196	1360	6	2021.05.05	435	1060895	1564	28616	142
11.06.2020	107	21182	237	1369	9	2021.05.06	436	1062527	1632	28710	94
12.06.2020	108	21404	222	1380	11	2021.05.07	437	1063949	1422	28799	89
13.06.2020	109	21679	275	1394	14	2021.05.08	438	1065254	1305	28903	104
14.06.2020	110	21999	320	1410	16	2021.05.09	439	1066111	857	28966	63
15.06.2020	111	22165	166	1427	17	2021.05.10	440	1066731	620	29034	68
16.06.2020	112	22415	250	1437	10	2021.05.11	441	1067887	1156	29135	101
17.06.2020	113	22760	345	1451	14	2021.05.12	442	1068817	930	29233	98
18.06.2020	114	23080	320	1473	22	2021.05.13	443	1069770	953	29308	75
19.06.2020	115	23400	320	1484	11	2021.05.14	444	1070605	835	29413	105
20.06.2020	116	23730	330	1500	16	2021.05.15	445	1071334	729	29485	72
21.06.2020	117	24045	315	1512	12	2021.05.16	446	1071899	565	29523	38
22.06.2020	118	24291	246	1523	11	2021.05.17	447	1072291	392	29571	48
23.06.2020	119	24505	214	1539	16	2021.05.18	448	1072972	681	29662	91
24.06.2020	120	24826	321	1555	16	2021.05.19	449	1073679	707	29716	54
25.06.2020	121	25286	460	1565	10	2021.05.20	450	1074297	618	29777	61
26.06.2020	122	25697	411	1579	14	2021.05.21	451	1074781	484	29826	49
27.06.2020	123	26022	325	1589	10	2021.05.22	452	1075236	455	29885	59
28.06.2020	124	26313	291	1612	23	2021.05.23	453	1075543	307	29941	56
2020.06.29	125	26582	269	1634	22	2021.05.24	454	1075773	230	29977	36
2020.06.30	126	26970	388	1651	17	2021.05.25	455	1076154	381	30040	63
2020.07.01	127	27296	326	1667	16	2021.05.26	456	1076533	379	30092	52
2020.07.02	128	27746	450	1687	20	2021.05.27	457	1076840	307	30131	39
2020.07.03	129	28166	420	1708	21	2021.05.28	458	1077161	321	30174	43
2020.07.04	130	28582	416	1731	23	2021.05.29	459	1077426	265	30247	73
2020.07.05	131	28973	391	1750	19	2021.05.30	460	1077584	158	30276	29
2020.07.06	132	29223	250	1768	18	2021.05.31	461	1077737	153	30312	36
2020.07.07	133	29620	397	1799	31	2021.06.01	462	1077978	241	30353	41

2020.07.08	134	30175	555	1817	18	2021.06.02	463	1078142	164	30415	62
2020.07.09	135	30789	614	1834	17	2021.06.03	464	1078338	196	30499	84
2020.07.10	136	31381	592	1847	13	2021.06.04	465	1078563	225	30612	113
2020.07.11	137	32079	698	1871	24	2021.06.05	466	1078742	179	30725	113
2020.07.12	138	32535	456	1884	13	2021.06.06	467	1078863	121	30815	90
2020.07.13	139	32948	413	1901	17	2021.06.07	468	1078952	89	30878	63
2020.07.14	140	33585	637	1931	30	2021.06.08	469	1079154	202	31155	277
2020.07.15	141	34226	641	1952	21	2021.06.09	470	1079294	140	31383	228
2020.07.16	142	35003	777	1971	19	2021.06.10	471	1079427	133	31531	148
2020.07.17	143	35802	799	1988	17	2021.06.11	472	1079530	103	31681	150
2020.07.18	144	36691	889	2009	21	2021.06.12	473	1079657	127	31804	123
2020.07.19	145	37458	767	2026	17	2021.06.13	474	1079726	69	31834	30
2020.07.20	146	38139	681	2038	12	2021.06.14	475	1079776	50	31861	27
2020.07.21	147	39133	994	2074	36	2021.06.15	476	1079879	103	31957	96
2020.07.22	148	40163	1030	2101	27	2021.06.16	477	1079983	104	32028	71
2020.07.23	149	41275	1112	2126	25	2021.06.17	478	1080070	87	32115	87
2020.07.24	150	42394	1119	2150	24	2021.06.18	479	1080140	70	32212	97
2020.07.25	151	43678	1284	2165	15	2021.06.19	480	1080203	63	32308	96
2020.07.26	152	44798	1120	2187	22	2021.06.20	481	1080256	53	32326	18
2020.07.27	153	45902	1104	2206	19	2021.06.21	482	1080282	26	32391	65
2020.07.28	154	47053	1151	2239	33	2021.06.22	483	1080323	41	32465	74
2020.07.29	155	48235	1182	2269	30	2021.06.23	484	1080389	66	32626	161
2020.07.30	156	49591	1356	2304	35	2021.06.24	485	1080457	68	32771	145
2020.07.31	157	50886	1295	2343	39	2021.06.25	486	1080522	65	32911	140
2020.08.01	158	52111	1225	2379	36	2021.06.26	487	1080584	62	33177	266
2020.08.02	159	53186	1075	2413	34	2021.06.27	488	1080630	46	33253	76
2020.08.03	160	54009	823	2432	19	2021.06.28	489	1080667	37	33311	58
2020.08.04	161	55241	1232	2480	48	2021.06.29	490	1080740	73	33605	294
2020.08.05	162	56550	1309	2521	41	2021.06.30	491	1080792	52	33786	181
2020.08.06	163	57895	1345	2566	45	2021.07.01	492	1080823	31	33861	75
2020.08.07	164	59273	1378	2616	50	2021.07.02	493	1080860	37	33898	37
2020.08.08	165	60623	1350	2659	43	2021.07.03	494	1080918	58	33925	27
2020.08.09	166	61768	1145	2700	41	2021.07.04	495	1080951	33	33928	3
2020.08.10	167	62547	779	2729	29	2021.07.05	496	1080979	28	33973	45
2020.08.11	168	63762	1215	2764	35	2021.07.06	497	1081030	51	34021	48
2020.08.12	169	65177	1415	2807	43	2021.07.07	498	1081090	60	34098	77
2020.08.13	170	66631	1454	2860	53	2021.07.08	499	1081120	30	34168	70
2020.08.14	171	68046	1415	2904	44	2021.07.09	500	1081174	54	34197	29
2020.08.15	172	69374	1328	2954	50	2021.07.10	501	1081210	36	34216	19
2020.08.16	173	70461	1087	2991	37	2021.07.11	502	1081236	26	34217	1
2020.08.17	174	71194	733	3029	38	2021.07.12	503	1081275	39	34219	2
2020.08.18	175	72208	1014	3074	45	2021.07.13	504	1081326	51	34233	14
2020.08.19	176	73617	1409	3106	32	2021.07.14	505	1081405	79	34242	9
2020.08.20	177	74963	1346	3154	48	2021.07.15	506	1081467	62	34245	3
2020.08.21	178	76355	1392	3196	42	2021.07.16	507	1081539	72	34250	5
2020.08.22	179	77544	1189	3233	37	2021.07.17	508	1081588	49	34252	2
2020.08.23	180	78505	961	3272	39	2021.07.18	509	1081632	44	34253	1
2020.08.24	181	79330	825	3309	37	2021.07.19	510	1081678	46	34254	1
2020.08.25	182	80390	1060	3367	58	2021.07.20	511	1081773	95	34258	4
2020.08.26	183	81646	1256	3421	54	2021.07.21	512	1081875	102	34260	2
2020.08.27	184	83150	1504	3459	38	2021.07.22	513	1081953	78	34264	4
2020.08.28	185	84468	1318	3507	48	2021.07.23	514	1082057	104	34266	2
2020.08.29	186	85833	1365	3539	32	2021.07.24	515	1082183	126	34267	1
2020.08.30	187	86785	952	3578	39	2021.07.25	516	1082292	109	34268	1
2020.08.31	188	87540	755	3621	43	2021.07.26	517	1082376	84	34270	2
2020.09.01	189	88593	1053	3681	60	2021.07.27	518	1082551	175	34273	3
2020.09.02	190	89891	1298	3721	40	2021.07.28	519	1082710	159	34274	1

2020.09.03	191	91256	1365	3765	44	2021.07.29	520	1082880	170	34275	1
2020.09.04	192	92595	1339	3812	47	2021.07.30	521	1083033	153	34278	3
2020.09.05	193	93864	1269	3850	38	2021.07.31	522	1083189	156	34281	3
2020.09.06	194	95014	1150	3893	43	2021.08.01	523	1083341	152	34286	5
2020.09.07	195	95897	883	3926	33	2021.08.02	524	1083478	137	34291	5
2020.09.08	196	97033	1136	3967	41	2021.08.03	525	1083711	233	34297	6
2020.09.09	197	98304	1271	4018	51	2021.08.04	526	1083982	271	34298	1
2020.09.10	198	99684	1380	4065	47	2021.08.05	527	1084226	244	34300	2
2020.09.11	199	101075	1391	4100	35	2021.08.06	528	1084456	230	34305	5
2020.09.12	200	102386	1311	4127	27	2021.08.07	529	1084711	255	34313	8
2020.09.13	201	103495	1109	4163	36	2021.08.08	530	1084919	208	34316	3
2020.09.14	202	104187	692	4185	22	2021.08.09	531	1085100	181	34319	3
2020.09.15	203	105298	1111	4236	51	2021.08.10	532	1085412	312	34323	4
2020.09.16	204	107011	1713	4285	49	2021.08.11	533	1085738	326	34331	8
2020.09.17	205	108690	1679	4312	27	2021.08.12	534	1086109	371	34334	3
2020.09.18	206	110217	1527	4360	48	2021.08.13	535	1086492	383	34337	3
2020.09.19	207	111550	1333	4402	42	2021.08.14	536	1086900	408	34342	5
2020.09.20	208	112781	1231	4435	33	2021.08.15	537	1087223	323	34348	6
2020.09.21	209	113589	808	4458	23	2021.08.16	538	1087509	286	34353	5
2020.09.22	210	114648	1059	4503	45	2021.08.17	539	1088053	544	34359	6
2020.09.23	211	116415	1767	4550	47	2021.08.18	540	1088594	541	34365	6
2020.09.24	212	118054	1639	4591	41	2021.08.19	541	1089189	595	34379	14
2020.09.25	213	119683	1629	4633	42	2021.08.20	542	1089817	628	34388	9
2020.09.26	214	121235	1552	4687	54	2021.08.21	543	1090408	591	34403	15
2020.09.27	215	122673	1438	4718	31	2021.08.22	544	1090925	517	34412	9
2020.09.28	216	123944	1271	4748	30	2021.08.23	545	1091340	415	34425	13
2020.09.29	217	125414	1470	4792	44	2021.08.24	546	1092122	782	34439	14
2020.09.30	218	127572	2158	4825	33	2021.08.25	547	1092971	849	34453	14
2020.10.01	219	129658	2086	4862	37	2021.08.26	548	1093924	953	34471	18
2020.10.02	220	132001	2343	4915	53	2021.08.27	549	1094870	946	34490	19
2020.10.03	221	134065	2064	4947	32	2021.08.28	550	1095885	1015	34509	19
2020.10.04	222	135900	1835	5003	56	2021.08.29	551	1096753	868	34528	19
2020.10.05	223	137491	1591	5048	45	2021.08.30	552	1097452	699	34539	11
2020.10.06	224	139612	2121	5121	73	2021.08.31	553	1098765	1313	34570	31
2020.10.07	225	142570	2958	5203	82	2021.09.01	554	1100208	1443	34591	21
2020.10.08	226	145700	3130	5247	44	2021.09.02	555	1101678	1470	34617	26
2020.10.09	227	148886	3186	5299	52	2021.09.03	556	1103198	1520	34650	33
2020.10.10	228	152403	3517	5358	59	2021.09.04	557	1104766	1568	34678	28
2020.10.11	229	155283	2880	5411	53	2021.09.05	558	1106008	1242	34689	11
2020.10.12	230	157352	2069	5467	56	2021.09.06	559	1107043	1035	34714	25
2020.10.13	231	160461	3109	5535	68	2021.09.07	560	1109076	2033	34762	48
2020.10.14	232	164477	4016	5601	66	2021.09.08	561	1111155	2079	34792	30
2020.10.15	233	168490	4013	5674	73	2021.09.09	562	1113381	2226	34871	79
2020.10.16	234	172516	4026	5749	75	2021.09.10	563	1115901	2520	34914	43
2020.10.17	235	176468	3952	5812	63	2021.09.11	564	1118549	2648	34961	47
2020.10.18	236	180388	3920	5872	60	2021.09.12	565	1120804	2255	34990	29
2020.10.19	237	182854	2466	5931	59	2021.09.13	566	1122653	1849	35036	46
2020.10.20	238	186254	3400	5996	65	2021.09.14	567	1126582	3929	35132	96
2020.10.21	239	191102	4848	6065	69	2021.09.15	568	1130586	4004	35215	83
2020.10.22	240	196004	4902	6163	98	2021.09.16	569	1135027	4441	35286	71
2020.10.23	241	201032	5028	6245	82	2021.09.17	570	1139505	4478	35359	73
2020.10.24	242	205793	4761	6318	73	2021.09.18	571	1144893	5388	35456	97
2020.10.25	243	209648	3855	6391	73	2021.09.19	572	1148710	3817	35514	58
2020.10.26	244	212492	2844	6470	79	2021.09.20	573	1152052	3342	35592	78
2020.10.27	245	217216	4724	6574	104	2021.09.21	574	1158841	6789	35721	129
2020.10.28	246	222559	5343	6681	107	2021.09.22	575	1165886	7045	35851	130
2020.10.29	247	229040	6481	6764	83	2021.09.23	576	1172981	7095	35964	113

2020.10.30	248	235586	6546	6867	103	2021.09.24	577	1180097	7116	36109	145
2020.10.31	249	241339	5753	6968	101	2021.09.25	578	1187773	7676	36230	121
2020.11.01	250	246663	5324	7067	99	2021.09.26	579	1194106	6333	36341	111
2020.11.02	251	250704	4041	7153	86	2021.09.27	580	1199761	5655	36450	109
2020.11.03	252	258437	7733	7273	120	2021.09.28	581	1210810	11049	36658	208
2020.11.04	253	267088	8651	7419	146	2021.09.29	582	1221636	10826	36865	207
2020.11.05	254	276802	9714	7540	121	2021.09.30	583	1233668	12032	37041	176
2020.11.06	255	287062	10260	7663	123	2021.10.01	584	1244555	10887	37210	169
2020.11.07	256	296999	9937	7793	130	2021.10.02	585	1257145	12590	37394	184
2020.11.08	257	303751	6752	7879	86	2021.10.03	586	1265827	8682	37544	150
2020.11.09	258	306991	3240	8009	130	2021.10.04	587	1274119	8292	37677	133
2020.11.10	259	314295	7304	8186	177	2021.10.05	588	1289156	15037	37929	252
2020.11.11	260	324094	9799	8389	203	2021.10.06	589	1303900	14744	38260	331
2020.11.12	261	334236	10142	8510	121	2021.10.07	590	1318367	14467	38542	282
2020.11.13	262	343725	9489	8684	174	2021.10.08	591	1332221	13854	38927	385
2020.11.14	263	353185	9460	8813	129	2021.10.09	592	1346240	14019	39209	282
2020.11.15	264	360281	7096	8926	113	2021.10.10	593	1356640	10400	39420	211
2020.11.16	265	365212	4931	9075	149	2021.10.11	594	1365788	9148	39629	209
2020.11.17	266	373474	8262	9261	186	2021.10.12	595	1382531	16743	40071	442
2020.11.18	267	383743	10269	9429	168	2021.10.13	596	1398264	15733	40461	390
2020.11.19	268	393851	10108	9596	167	2021.10.14	597	1414647	16383	40765	304
2020.11.20	269	403123	9272	9756	160	2021.10.15	598	1430475	15828	41130	365
2020.11.21	270	412808	9685	9916	160	2021.10.16	599	1445714	15239	41482	352
2020.11.22	271	418645	5837	10047	131	2021.10.17	600	1457260	11546	41781	299
2020.11.23	272	422852	4207	10177	130	2021.10.18	601	1467401	10141	42042	261
2020.11.24	273	430605	7753	10373	196	2021.10.19	602	1486264	18863	42616	574
2020.11.25	274	440344	9739	10541	168	2021.10.20	603	1503422	17158	43039	423
2020.11.26	275	449349	9005	10712	171	2021.10.21	604	1519532	16110	43487	448
2020.11.27	276	457848	8499	10884	172	2021.10.22	605	1534942	15410	43844	357
2020.11.28	277	465982	8134	11045	161	2021.10.23	606	1550203	15261	44290	446
2020.11.29	278	471536	5554	11193	148	2021.10.24	607	1561928	11725	44679	389
2020.11.30	279	475362	3826	11331	138	2021.10.25	608	1571115	9187	44980	301
2020.12.01	280	479634	4272	11530	199	2021.10.26	609	1587880	16765	45503	523
2020.12.02	281	484550	4916	11665	135	2021.10.27	610	1602830	14950	46015	512
2020.12.03	282	492211	7661	11876	211	2021.10.28	611	1616027	13197	46430	415
2020.12.04	283	500273	8062	12052	176	2021.10.29	612	1628501	12474	46911	481
2020.12.05	284	508345	8072	12186	134	2021.10.30	613	1640607	12106	47324	413
2020.12.06	285	513576	5231	12320	134	2021.10.31	614	1648031	7424	47751	427
2020.12.07	286	517236	3660	12447	127	2021.11.01	615	1655024	6993	48073	322
2020.12.08	287	524675	7439	12660	213	2021.11.02	616	1666097	11073	48664	591
2020.12.09	288	532040	7365	12821	161	2021.11.03	617	1676293	10196	49115	451
2020.12.10	289	539107	7067	12948	127	2021.11.04	618	1685264	8971	49604	489
2020.12.11	290	545567	6460	13116	168	2021.11.05	619	1693532	8268	50087	483
2020.12.12	291	551900	6333	13264	148	2021.11.06	620	1701589	8057	50482	395
2020.12.13	292	556335	4435	13385	121	2021.11.07	621	1706882	5293	50755	273
2020.12.14	293	559587	3252	13494	109	2021.11.08	622	1711137	4255	50996	241
2020.12.15	294	565758	6171	13698	204	2021.11.09	623	1718726	7589	51483	487
2020.12.16	295	571749	5991	13862	164	2021.11.10	624	1725017	6291	51888	405
2020.12.17	296	577446	5697	13969	107	2021.11.11	625	1730433	5416	52206	318
2020.12.18	297	582786	5340	14157	188	2021.11.12	626	1735277	4844	52513	307
2020.12.19	298	587944	5158	14296	139	2021.11.13	627	1739283	4006	52836	323
2020.12.20	299	591294	3350	14394	98	2021.11.14	628	1742304	3021	53069	233
2020.12.21	300	593783	2489	14481	87	2021.11.15	629	1744440	2136	53264	195
2020.12.22	301	598792	5009	14636	155	2021.11.16	630	1748568	4128	53661	397
2020.12.23	302	604251	5459	14766	130	2021.11.17	631	1752103	3535	54011	350
2020.12.24	303	608561	4310	14912	146	2021.11.18	632	1755179	3076	54343	332
2020.12.25	304	612373	3812	15023	111	2021.11.19	633	1758068	2889	54624	281

2020.12.26	305	613760	1387	15108	85	2021.11.20	634	1760765	2697	54838	214
2020.12.27	306	615809	2049	15230	122	2021.11.21	635	1762701	1936	54959	121
2020.12.28	307	618429	2620	15334	104	2021.11.22	636	1764251	1550	55113	154
2020.12.29	308	623066	4637	15469	135	2021.11.23	637	1766987	2736	55386	273
2020.12.30	309	627941	4875	15596	127	2021.11.24	638	1769783	2796	55617	231
2020.12.31	310	632263	4322	15767	171	2021.11.25	639	1771887	2104	55829	212
2021.01.01	311	636201	3938	15841	74	2021.11.26	640	1773845	1958	55989	160
2021.01.02	312	637395	1194	15919	78	2021.11.27	641	1775572	1727	56169	180
2021.01.03	313	640429	3034	15979	60	2021.11.28	642	1776949	1377	56275	106
2021.01.04	314	643559	3130	16057	78	2021.11.29	643	1778045	1096	56382	107
2021.01.05	315	648288	4729	16178	121	2021.11.30	644	1779667	1622	56529	147
2021.01.06	316	654007	5719	16299	121	2021.12.01	645	1780808	1141	56618	89
2021.01.07	317	658958	4951	16410	111	2021.12.02	646	1781957	1149	56684	66
2021.01.08	318	663799	4841	16506	96	2021.12.03	647	1783630	1673	56890	206
2021.01.09	319	668202	4403	16592	86	2021.12.04	648	1785120	1490	57021	131
2021.01.10	320	671284	3082	16654	62	2021.12.05	649	1786036	916	57099	78
2021.01.11	321	673271	1987	16725	71	2021.12.06	650	1786839	803	57153	54
2021.01.12	322	676968	3697	16881	156	2021.12.07	651	1788260	1421	57260	107
2021.01.13	323	681392	4424	16969	88	2021.12.08	652	1789539	1279	57360	100
2021.01.14	324	684917	3525	17035	66	2021.12.09	653	1790571	1032	57434	74
2021.01.15	325	688270	3353	17098	63	2021.12.10	654	1791502	931	57531	97
2021.01.16	326	691488	3218	17164	66	2021.12.11	655	1791502	0	57531	0
2021.01.17	327	693644	2156	17221	57						

*Additional data collected after this investigation [C], we hope it will be useful for those who intend to do further work.

In this work we will try to investigate only the first wave of Covid-19 spread as delimited and shownin Figure S1.

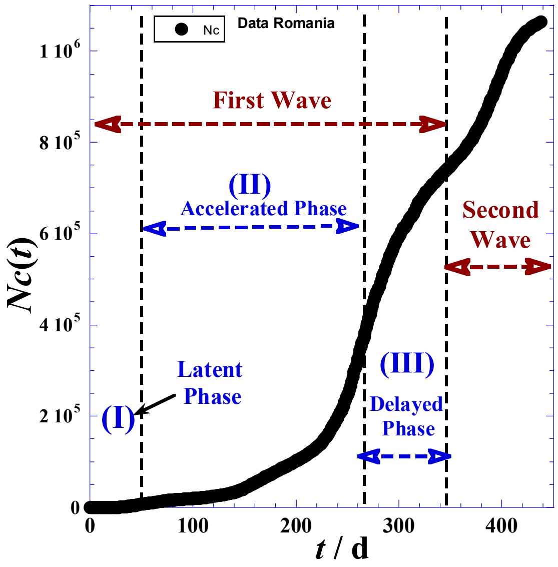

Figure S1.Total Reported Cases for the First 440 days of the Pandemic in Romania.

2S. Elimination of Duplicated Data

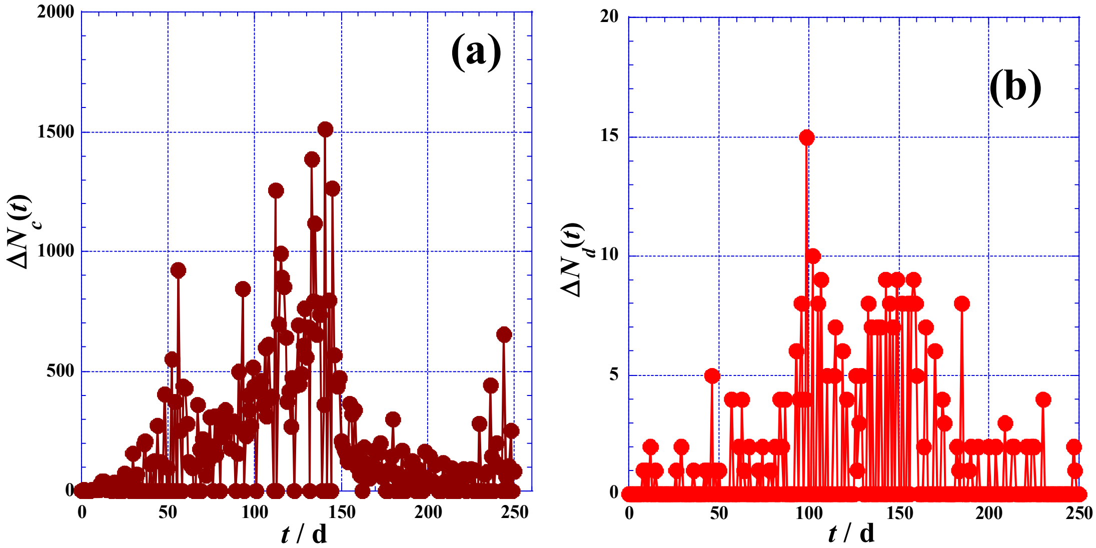

Figure S2. New daily reported cases (a) and new daily deaths (b) for the eight months of the pandemic in Romania (Reported from original data without smoothing of duplicated values).

Before investigations and data handling, we observe a certain difference in how countries record COVID-19 data due to the absence of accepted international standard for how they measure this information. In this context, this reflects the presence of duplicate information in the Excel sheet that is why we have suggested the flowchart for adjustment this duplicate data. We add that the several null values of new daily reported cases and deaths observed in the (Fig. S2) along the whole range of time) are due to the duplications of data. We assume the index (i) is always the first duplicate data.

- (i-1) the day before the duplicate data.
- (i+1) is the day after the first duplicate data.
- We don't consider more than triple duplicate data.

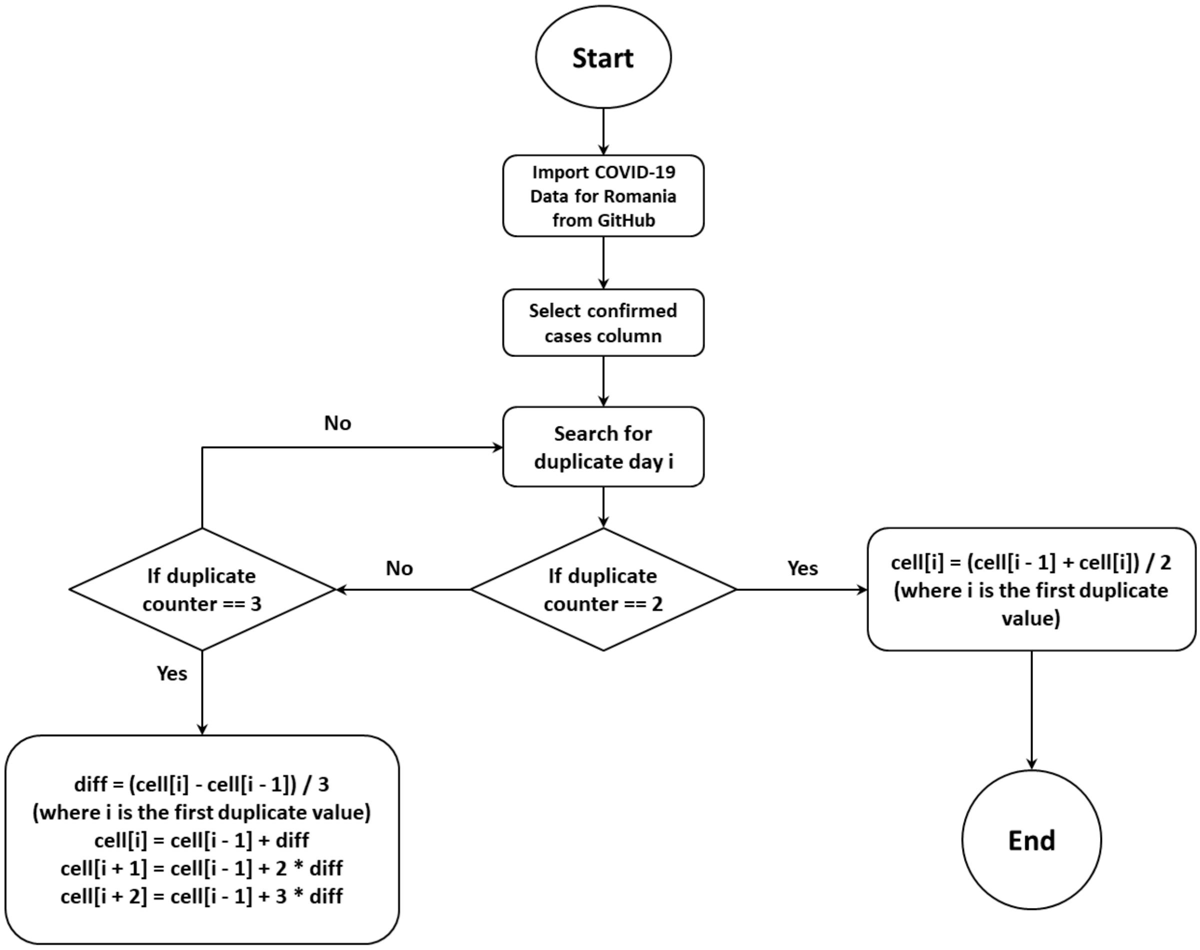

Scheme S1. Flowchart for Adjustment the Duplicate Data in Romania Excel Sheet.

3S.Determining Intervals of Concavity and Inflection Points

The tangents'techniqueimplicates the use of graphical method in determining the inflection points. The method starts with drawing three straight lines: The first two lines referred to as the parallel tangents which are tangential to the curve on either side of the jump of *Nc* (or *Nd*), the third line is parallel to aforementioned tangents and equidistant to each of them.The third line will approximately tend to intersect the curve at the midpoint of the jump of *Nc* (or *Nd*), where the coordinates of this intersection point (Figs. S3 and S4) will correspond to the coordinates of the inflection point Fc(t_{c1},Nc_1) or Fd(t_{d1},Nd_1).

It is perceptible that the inflection point is the point where there is a change in concavity (Fig. 1). The method of determining the intervals of concavity is same as the method used to determine the intervals of increase.decrease as shown in Fig. 2, except that the second derivative is used in place of the first derivative (Fig. 4S). More precisely,as$d(dN.dt).dt=d^2N.dt^2$, the intervals of increase.decrease for the first derivative in Fig. 2 will determine the concavity of $N(t)$. The two extrema of Figure S4 indicate approximately, where the two tangent lines are positioned as optimal positions.

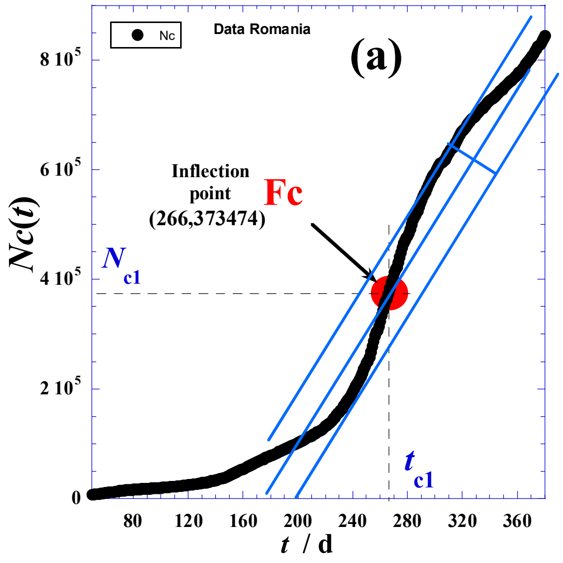

Figure S3a.Graphical determination of the inflection point Fc(t_{c1},N_{c1}) related the total reported cases *Nc*(*t*) for the first 350 days in Romania.

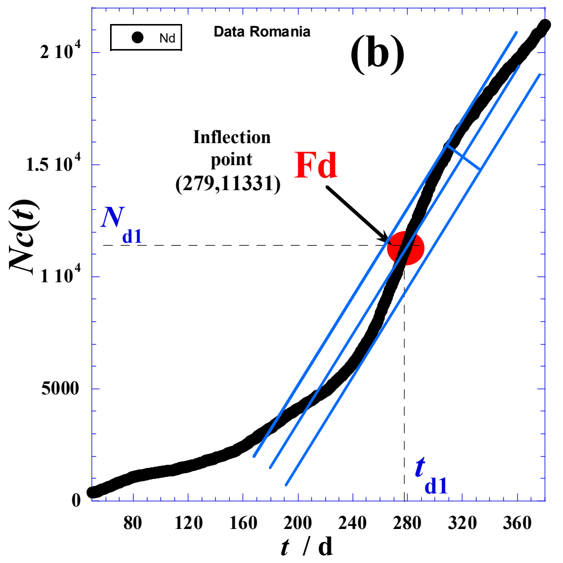

Figure S3b. Graphical determination of the inflection point Fd(t_{d1},N_{d1}) related the total death cases *Nd*(*t*) for the first 350 days in Romania.

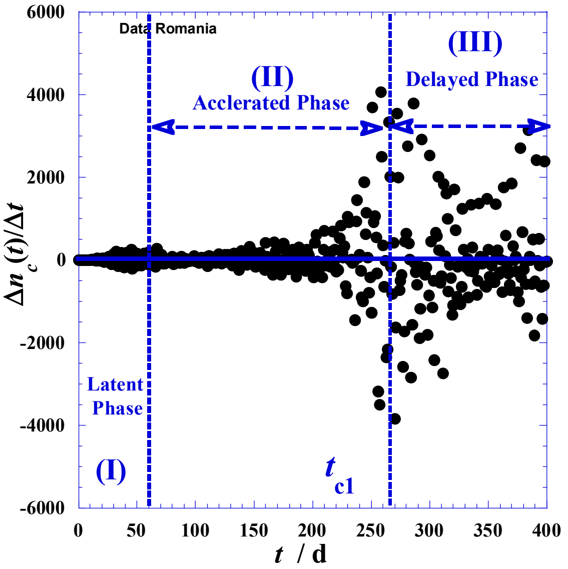

Figure S4.Seconddderivative function ($d^2Nc.dt^2$) related the total death cases $Nc(t)$ for the first 400 days in Romania.

Here, there are some the aberrant values (with opposite sign) due to fluctuations and double derivative operations. In addition, the existence of some small waves disturbs the trend of points

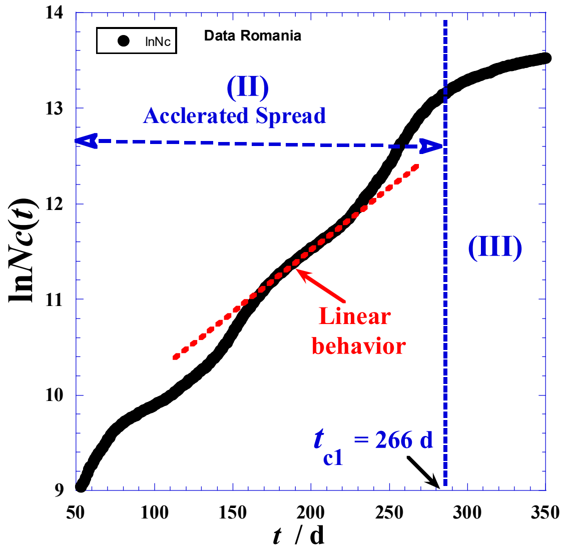

Figure S5.Naturallogarithm of total reported cases ln$Nc(t)$ with time in the accelerated phase (II).

Note, we observe other zones of linear behavior in the center of accelerated phase, because there are secondary small waves indicated by the Figure 3.

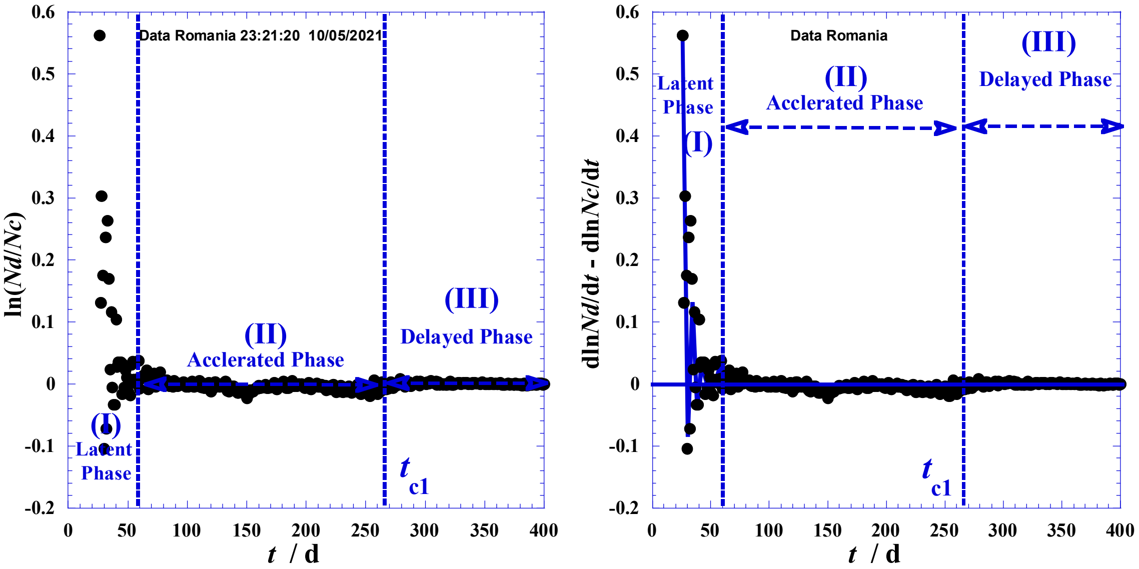

Figure S6. Difference between logarithms of the derivative functions of the total reported cases and deaths; to confirm the global maximum occurring In*Nc*.*Nd*-Curve (Figure 5).

Due to perturbations in data of scatter points, we cannot see clearlywhere this function cuts the time axis. Nevertheless, we are sure that on approximately the time ($t = t_{c1}$) must cuts the zero for the highly day illustrated by the inflection point in Fig. 1 and a maximum in Fig. 2. In the same context, we can predict the final value of the ratio Nd.Nc of Fig. 5 in the case of symmetric behavior by the following expression:

$$\lim_{\infty} \frac{N_d(t)}{N_c(t)} = \frac{N_{d0}}{N_{c0}} e^{\frac{t_c}{\tau_c} - \frac{t_d}{\tau_d}} \quad \text{(S1)}$$
